# Proenkephalin 119–159 in Heart Failure: From Pathophysiology to Clinical Implications

**DOI:** 10.3390/jcm14082657

**Published:** 2025-04-13

**Authors:** Dionysis Matsiras, Ioannis Ventoulis, Christos Verras, Vasiliki Bistola, Sofia Bezati, Barbara Fyntanidou, Effie Polyzogopoulou, John T. Parissis

**Affiliations:** 1 Department of Emergency Medicine, Attikon University Hospital, National and Kapodistrian University of Athens, Rimini 1, 12462 Athens, Greece; christos.verras@gmail.com (C.V.); vasobistola@yahoo.com (V.B.); sofiabezati@gmail.com (S.B.); effiepol@med.uoa.gr (E.P.); jparissis@yahoo.com (J.T.P.); 2 Department of Occupational Therapy, University of Western Macedonia, Keptse Area, 50200 Ptolemaida, Greece; iventoulis@uowm.gr; 3 Department of Emergency Medicine, AHEPA University Hospital, Aristotle University of Thessaloniki, St. Kiriakidi 1, 54636 Thessaloniki, Greece; bfyntan@yahoo.com

**Keywords:** proenkephalin 119–159, enkephalins, heart failure, worsening renal function, pathophysiology, opioid peptide receptors

## Abstract

Heart failure (HF) is a challenging clinical syndrome with high morbidity and mortality rates. Along the spectrum of cardiovascular diseases, HF constitutes an ever-expanding area of research aiming at combating the associated mortality and improving the prognosis of patients with HF. Although natriuretic peptides have an established role among biomarkers in HF diagnosis and prognosis, several novel biomarkers reflecting the complex pathophysiology of HF are under investigation for their ability to predict adverse clinical outcomes in HF. Proenkephalin 119–159 (PENK_119–159_) is a non-functional peptide belonging to the enkephalin family of the endogenous opioid system and is considered a surrogate biomarker of the biologically active enkephalin peptides. PENK_119–159_ has demonstrated promising results in predicting short- and long-term mortality, readmission rates, and worsening renal function in patients with HF. Indeed, in the setting of HF, the levels of both active enkephalins and their surrogate PENK_119–159_ are elevated and are associated with a dismal prognosis. However, the biological effects of PENK_119–159_ remain largely unknown. Thus, it is crucial to gain a deeper insight into both the physiology of the enkephalin peptide family and the enkephalin-mediated cardiovascular regulation. In order to elucidate the complex pathophysiological mechanisms that lead to the upregulation of enkephalins in patients with HF, as well as the potential clinical implications of elevated enkephalins and PENK_119–159_ levels in this patient population, the present review will describe the physiology and distribution of the endogenous opioid peptides and their corresponding opioid receptors, with a particular focus on the action of enkephalins. The effects of the enkephalin peptides will be analyzed in both healthy subjects and patients with HF, especially with regard to their role in the regulation of cardiovascular and renal function. The review will also discuss the findings of recent studies that have explored the prognostic value of PENK_119–159_ in patients with HF.

## 1. Introduction

Heart failure (HF) is a chronic and progressive clinical syndrome that portends a poor prognosis since it is characterized by recurrent hospitalizations superimposed on a gradually deteriorating clinical course [1]. In an attempt to further reduce HF-associated morbidity and mortality, the use of several biomarkers has been advocated for the assessment of HF severity, risk stratification, and prognosis. Recent literature has shed light on a so far neglected biomarker, proenkephalin 119–159 (PENK_119–159_), which belongs to the enkephalin family of endogenous opioid peptides (EOPs) and has been shown to predict adverse outcomes in patients with HF [2].

Associations between enkephalins and HF were initially demonstrated during the 1980s, predominantly in preclinical models of HF. Later on, the discovery of novel measurable peptides, such as PENK_119–159_, reignited interest in investigating the relationship between the enkephalinergic system and HF and extended clinical observations in human subjects. PENK_119–159_, a biologically inert peptide, has lately emerged as a surrogate biomarker of mature (biologically active) enkephalins owing to its long half-life of approximately 48 h after sample collection, which renders it a more stable molecule in vitro [3]. Given the fact that it is renally excreted through glomerular filtration, it has been proposed as a prognostic marker of kidney injury [4]. Recent studies also suggest that PENK_119–159_ predicts short- and long-term mortality, HF readmission rates, and the occurrence of cardiorenal syndrome in patients with HF [2]. The excitement of these findings, though, is blunted by the limited number of studies, as well as by their observational nature, which precludes any causal associations to be made between PENK_119–159_ and prognostic endpoints.

Nevertheless, a larger body of evidence concerning the effects of active EOPs on cardiovascular function reveals that enkephalin peptides and opioid receptors (ORs) are expressed in both failing and non-failing myocardial tissue [5,6,7]. It is therefore not surprising that OR agonism or blockade may generate certain cardiovascular responses [8]. This review will primarily unravel basic aspects regarding the production and distribution of EOPs, the cardiovascular and renal effects of the enkephalinergic system under physiological conditions, and the upregulation of enkephalins in HF. By bridging the gap between pathophysiological mechanisms and clinical implications, this review aims to provide a deeper understanding of the association between plasma PENK_119–159_ levels and clinical outcomes in patients with HF.

## 2. Enkephalins

### 2.1. Endogenous Opioid Peptides

It was not until the mid-1970s that the utilization of specific functional bioassays led to the identification of the first EOPs, termed enkephalins, in the brain tissue of guinea pigs. Subsequent research allowed the discovery of various peptides, all of which shared a common N-terminal amino acid sequence and were collectively coined EOPs. EOPs were then assorted into three main groups: enkephalins, endorphins, and dynorphins, which derive from the prohormones proenkephalin, proopiomelanocortin, and pro-dynorphin, respectively. Each precursor molecule does not generate peptides on a 1:1 ratio but can rather be seen as a long amino acid sequence from which various peptides arise. Owing to their similar amino acid sequences, peptides of all three families can act as ligands of μ (mu)-, κ (kappa)-, and δ (delta)-ORs. Of note, each peptide exhibits varying affinity for each corresponding type of OR, thereby giving rise to complex peptide receptor signaling [9].

### 2.2. Enkephalin Peptides

**Figure 1:**
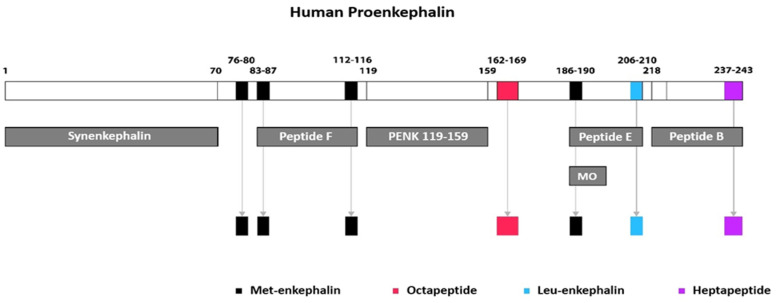
Human proenkephalin peptide processing. The 243-amino-acid-long proenkephalin is cleaved at different sites to yield the intermediate-sized peptides Synenkephalin (1–70), peptide F (83–116), PENK 119–159, peptide E (186–210), and peptide B (218–243), as well as the small-sized biologically active peptides Met^5^-enkephalin (76–80) and Octapeptide (162–169). Further cleavage of intermediate peptides generates a larger number of active peptides, namely two copies of Met^5^-enkephalin (83–87) and (112–116) from peptide F; one Met^5^-enkephalin (186–190) from sequential processing of peptide E into metorphamide (MO); one Leu^5^-enkephalin (206–210) directly from peptide E; and finally, one heptapeptide (237–243) from peptide B. Collectively, one human proenkephalin molecule gives rise to active enkephalin peptides at a ratio of 4 Met^5^-enkephalin: 1 Leu^5^-enkephalin: 1 heptapeptide: 1 octapeptide [3,10,11,12,13,14].

Enkephalin is an umbrella term encompassing all proenkephalin-derived peptides, albeit frequently misconceived as two specific peptides, methionine (Met)- and leucine (Leu)-enkephalin. Human proenkephalin is a 243-amino-acid-long prohormone that stems from preproenkephalin (a preprohormone consisting of 267 amino acids) after proteolytic cleavage of the N-terminal signal sequence by a specific signal peptidase [3]. Human proenkephalin serves as the precursor molecule that undergoes post-translational processing by prohormone convertases to eventually yield biologically active, short amino acid peptides, all of which share a common N-terminal sequence (Tyr^1^-Gly^2^-Gly^3^-Phe^4^) and are the following: 4 copies of Met^5^-enkephalin, 1 copy of Leu^5^-enkephalin, and 2 different C-terminal extended Met-enkephalin peptides, namely a hepta-peptide (MEAP: Met^5^-enkephalin-Arg^6^-Phe^7^), and an octa-peptide (MEAGL: Met^5^-enkephalin-Arg^6^-Gly^7^-Leu^8^) [3,10]. Active enkephalin peptides are generated either immediately upon the first cleavage of proenkephalin or at later stages from further processing of intermediate peptides, which have been termed peptide B (or enkelytin), peptide E, and peptide F [11,12]. Peptides F and B are further cleaved to generate 2 Met-enkephalins and 1 heptapeptide (MEAP), respectively, whereas peptide E is cleaved to 1 Leu-enkephalin and 1 Met-enkephalin, after being processed to the intermediate metorphamide [13]. During this proteolytic process, certain peptide fragments are generated as by-products from the cleavage of proenkephalin and presumably remain inactive after cleavage. These inactive peptides are Synenkephalin and PENK_119–159_ and contain specific amino acid sequences of the prohormone. In particular, PENK_119–159_ has a length of 41 amino acids (119–159) [3], while Synenkephalin represents a 70-amino-acid-long peptide [14]. The sequential processing of proenkephalin into intermediate- and small-sized peptides is schematically depicted in Figure 1.

Enkephalin synthesis, once thought to occur solely in the central nervous system (CNS) and adrenal medulla, has now been shown to take place in various tissues, including the myocardium. Upon synthesis, enkephalins are either stored intracellularly or secreted, depending on each tissue’s storage capacity [10]. Under normal conditions, human enkephalins circulate in plasma at constant levels throughout the day, lacking circadian rhythmicity [15]. Measurement of the plasma levels of mature enkephalins is cumbersome because they exhibit a very short (15 min) in vitro half-life [3]. Nevertheless, biologically active enkephalin peptides bind to ORs with peptide-specific affinity and generate a certain response. Although it is frequently mentioned that each EOP family selectively binds to a specific OR (enkephalins to δ-ORs, endorphins to μ-ORs, and dynorphins to κ-ORs), studies reveal that the receptor affinity is peptide-specific and not family-specific. Met-enkephalin and Leu-enkephalin bind to all ORs with a higher affinity for δ-receptors, while heptapeptide, octapeptide, and Met-enkephalin’s precursor peptides, such as metorphamide, display equal or higher affinity for μ- and κ-receptors [9].

### 2.3. Opioid Peptide Receptors in Myocardium

Expression of ORs in the heart has long been demonstrated in humans [7,10,16], indicating that the myocardium is under opioid regulation, at least to some extent. Sobanski et al. reported the expression of all ORs in human cardiac tissue obtained post-mortem. ORs were expressed in both cardiomyocytes and nerve fibers innervating the heart and demonstrated a specific pattern of distribution in the human myocardium. A higher degree of μ-OR immunoreactivity was observed in cardiomyocytes of the inferior left ventricular (LV) wall, while κ-ORs were preferentially expressed in myocardial cells of the right ventricle and the interventricular septum, and δ-ORs in right ventricular myocytes [7].

Apart from their cardiac expression, the anatomical location of ORs may suggest their biological role in cardiovascular regulation. The colocalization of δ-ORs with sympathetic, parasympathetic, and sensory fibers within the atrial cells of neonatal rats indicates an autonomic nervous system (ANS)-mediated cardiovascular regulation by the EOP system [5]. In LV rat tissue, δ-ORs, κ-ORs, and μ-ORs are present in the transverse tubules (T-tubules) in close proximity with both the dihydropyridine long transient calcium channels (LTCC) of the myocardial plasma membrane and the ryanodine receptors located on the sarcoplasmic reticulum (SR) membrane. This reinforces the hypothesis that OR stimulation modulates intracellular calcium homeostasis and excitation–contraction coupling, thus resulting in inotropic changes [5,6,17]. Overall, these findings suggest that OR stimulation is involved in cardiomyocyte function and afferent sensory signaling. Furthermore, μ-ORs and κ-ORs are present on the mitochondrial membrane, which implies a link between OR stimulation on the one hand and phosphorylation processes and anti-oxidation on the other [6,17].

### 2.4. Enkephalins in Myocardial Tissue

Enkephalin pentapeptides have been identified in cardiac extracts from guinea pigs, particularly in the terminal nerve fibers of cardiac ganglia. Interestingly enough, each Leu-enkephalin peptide corresponded to four Met-enkephalins, similar to the 1:4 ratio of the pentapeptides produced by proenkephalin cleavage. What is more, cardiac sympathectomy resulted in a 99% and 70% reduction in the cardiac concentrations of norepinephrine and enkephalins, respectively. This observation led to the presumption that a large enkephalin reservoir was present within the cardiac nerve fibers of the sympathetic nervous system (SNS), while a smaller extraneural production site also existed, which was responsible for the production of the remaining 30% of enkephalins [18]. Likewise, in humans, Rechardt et al. reported the presence of Leu-enkephalin, alongside other non-opioid peptides, in cholinergic and adrenergic nerve terminals located within the right atrial tissue [19].

The presence of enkephalins in the heart is not limited solely to the ANS fibers, but it also extends to the cardiomyocytes themselves. Proenkephalin mRNA is evident in rodent and human cardiomyocytes. However, the concentrations of enkephalin peptides in the ventricular tissue were found to be disproportionately low compared to the high amounts of proenkephalin mRNA detected in the same tissue. Based on this initial observation, the translational potential of proenkephalin mRNA in the ventricles was questioned. The discrepancy between abundant amounts of proenkephalin mRNA and low concentrations of enkephalin peptides in the ventricular tissue was attributed either to a selective inability of the ventricular proenkephalin mRNA to be translated due to inhibitory factors or to the absence of secretory vesicles in the rat ventricles resulting in a rapid turnover of enkephalin peptides [10]. Indeed, in contrast to atria, ventricles lack storage granules, wherein enkephalins can be stored until released [20]. Therefore, it is postulated that the ventricles exhibit differences in the processing, storage, and release of enkephalins, which presumably may be immediately synthesized and directly released from the ventricles into the circulation and are thus scarcely found in the ventricular tissue itself. Another hypothesis suggests that enkephalins can reside within the myocardial tissue in non-pentapeptide forms, such as intermediate-sized peptides and MEAP. Indeed, Younes et al. isolated different-sized peptides based on their molecular weight and reported that, in isolated normoxic working rat hearts, there were 10-fold higher contents of peptide B and MEAP than Met-enkephalin. Surprisingly, the principal enkephalin peptide released in the coronary effluent was Met-enkephalin and not peptide B or MEAP [21].

Based on these studies, the presence of a local enkephalinergic pool in cardiac ANS fibers, as well as the existence of a myocardial pool with a potential of enkephalin synthesis, are more than evident [10,19]. Low concentrations of enkephalins observed in the cardiac tissue of other mammals could be explained by two plausible mechanisms. The first one involves proenkephalin cleavage and immediate secretion of its mature peptides into the circulation without them being stored in secretory granules, thus rendering them undetectable in the cardiac tissue. The second one entails selective proenkephalin cleavage into intermediate enkephalins, which are subsequently stored as intermediate peptides and MEAP, while the rest of the prohormone either remains un-cleaved or is directly secreted.

### 2.5. Enkephalins in Adrenal Tissue

In addition to the neural and myocardial enkephalinergic expression, there is another major peripheral storage and secretory pool that lies within the adrenal glands. Cross-sections of human adrenal glands reveal high contents of Met-enkephalin in the adrenal medulla [15], which seem to be stored in granules alongside catecholamines and are co-released upon neural stimulation [22]. Furthermore, blood samples obtained from the adrenal vein reveal high plasma Met-enkephalin levels when compared to the ipsilateral femoral vein sample, supporting the hypothesis that the human adrenal medulla constitutes a major enkephalinergic reservoir [15].

## 3. Enkephalin-Mediated Cardiovascular Regulation

### 3.1. CNS-Mediated Regulation

The complexity of the CNS-mediated cardiovascular regulation is demonstrated by the coexistence of both pressor and depressor areas in close proximity to each other, which lie at several distinct loci, such as the hypothalamus or the brainstem, and produce opposite hemodynamic responses upon stimulation by the same enkephalin analog. With regard to the cardiovascular center located in the medulla oblongata, it has been observed that the central cardiovascular effects of enkephalins are receptor-specific. When an enkephalin analog highly selective for μ-OR was injected into the nucleus ambiguous, it elicited a pressor response. On the other hand, selective κ-OR agonism led to depressor effects. Considering the fact that there is imaging evidence of abundant μ-OR and κ-OR in the brainstem nuclei, it could be deduced that the cardiovascular center in the medulla oblongata exerts its cardiovascular stimulatory or inhibitory effects upon μ-OR or κ-OR activation, respectively [8].

Physiologically, the cardiovascular center in the medulla oblongata controls heart contractility, heart rate, and vascular tone via ANS regulation. Although its components receive various extracardiac inputs, the review will focus on the neural circuit, which extends from the heart to the brain and vice versa. Afferent fibers from cardiac sensory nerves are mostly parasympathetic and travel through the vagus nerve to transmit signals mainly to the nucleus tractus solitarius (NTS), while a minority of them are sympathetic and, through projection to the thoracic dorsal root ganglion and the dorsal horn of the spinal cord, ascend to the brainstem, thalamus, and hypothalamus. Meanwhile, input from arterial baroreceptors and chemoreceptors is carried via cranial nerves IX and X to the NTS. Through complex interconnections between different nuclei, integration of the input finally transforms into a response via the efferent ANS fibers. Sympathetic and parasympathetic fibers descend through the spinal cord and the vagus nerve, respectively, to reach the heart, where they exert positive or negative inotropy and chronotropy. The efferent sympathetic fibers descend to the intermediolateral nucleus located at the lateral gray column of the thoracic spinal cord and exit through anterior roots to reach the cervicothoracic sympathetic chain, including the stellate ganglia, which finally innervate the heart through post-ganglionic sympathetic fibers. Meanwhile, efferent parasympathetic fibers originating from the medulla oblongata are carried through the vagus nerve as preganglionic fibers to the myocardium, where intracardiac ganglia serve as their first relay center. Both components of the ANS descend as preganglionic myelinated fibers until they reach the corresponding ganglion and then continue as demyelinated post-ganglionic fibers down to the nerve terminals, where they release their neurotransmitters to the synaptic cleft, namely norepinephrine or acetylcholine for sympathetic or parasympathetic nerve transmission, respectively [23]. Additionally, sympathetic preganglionic fibers reaching the adrenal glands are considered to stimulate the co-release of catecholamine and enkephalins from the adrenal medulla [22].

### 3.2. Presynaptic Modulation

Enkephalins are present in both adrenergic and cholinergic cardiac nerve terminals [10,18,19] and modulate neurotransmitter release [24].

With regard to adrenergic cardiac nerve terminals, it has been shown that enkephalins exert sympatholytic effects by inhibiting norepinephrine release. Administration of Leu-enkephalin in previously sympathetically stimulated canine sinoatrial nodal cells attenuated the adrenergic tachycardia, presumably via a κ-OR-mediated reduction in presynaptic catecholamine release. Although Leu-enkephalin is primarily a δ-OR agonist, the observed sympatholytic effect was likely driven by its milder κ-OR affinity since only κ-OR antagonism, but not δ-OR or μ-OR antagonism, was capable of completely abolishing the negative chronotropic effect of Leu-enkephalin [25].

On the other hand, presynaptic cardiac cholinergic modulation is a more complex phenomenon since OR activation may either inhibit or augment acetylcholine release in post-ganglionic parasympathetic nerve terminals. In regard to OR-mediated inhibitory effects, cholinergic nerve terminals possess δ-OR, which, upon enkephalinergic stimulation, attenuates presynaptic acetylcholine release [26]. Indeed, isolated atria of rabbits, rats, and guinea pigs with intact cholinergic intramural nerve fibers were exposed to enkephalin analogs both in resting states and upon cholinergic stimulation, while chronotropy, inotropy, and presynaptic neurotransmitter release were monitored. It was found that enkephalin analogs inhibited the cholinergic negative chronotropic effect in rabbits and rats but not in guinea pigs, exhibiting interspecies variations. By means of neurotransmitter radioactive labeling, it was further demonstrated that the cholinergic inhibition was mediated by decreased presynaptic acetylcholine release, while it was abolished by the OR antagonist naloxone. It is noteworthy that the administration of enkephalin analogs exerted inhibitory effects only upon cholinergic nerve stimulation, whereas it had no impact on resting conditions or when exogenous acetylcholine was introduced to the atrial tissue [24]. Moreover, Musha et al. evaluated the in vivo effects of Leu-enkephalin, Met-enkephalin, and morphine in sedated dogs both after electrical stimulation of the vagus nerve and after intracoronary administration of a direct muscarinic agonist, metacholine, which bypasses acetylcholine neurotransmission. Enkephalins inhibited vagal-induced bradycardia but had no effect on metacholine-induced bradycardia, in accordance with the hypothesis of presynaptic inhibition of acetylcholine release [26].

Apart from the aforementioned vagolytic effects of the enkephalins, other investigators have shown that MEAP exerted both vagolytic and vagotonic effects when administered into the interstitium of canine sinoatrial nodes during vagal stimulation. In detail, administration of MEAP in low doses led to vagotonic responses, whereas high doses of MEAP induced vagolytic responses. This may be explained by the bimodal nature of the δ-ORs located in the presynaptic terminal. Since both vagolytic and vagotonic responses were eliminated by δ-OR antagonism, it was hypothesized that two different receptors existed within the δ-OR family [27]. Based on this hypothesis, which stems from experimental studies on sensory neurons, δ1-OR is a Gs protein-coupled OR that augments acetylcholine release and improves vagal transmission, whereas δ2-OR is a Gi protein-coupled OR exerting the opposite effects [27,28]. Indeed, Western blot analysis has revealed the presence of two different δ-OR bands on presynaptic parasympathetic nerve terminals from canine cardiac tissue [29]. Within this context, the vagolytic effect exerted by high doses of MEAP is mediated by the activation of δ2-ORs, whereas lower doses of MEAP exert a vagotonic action and enhance cholinergic bradycardia by acting on δ1-ORs.

**Figure 2:**
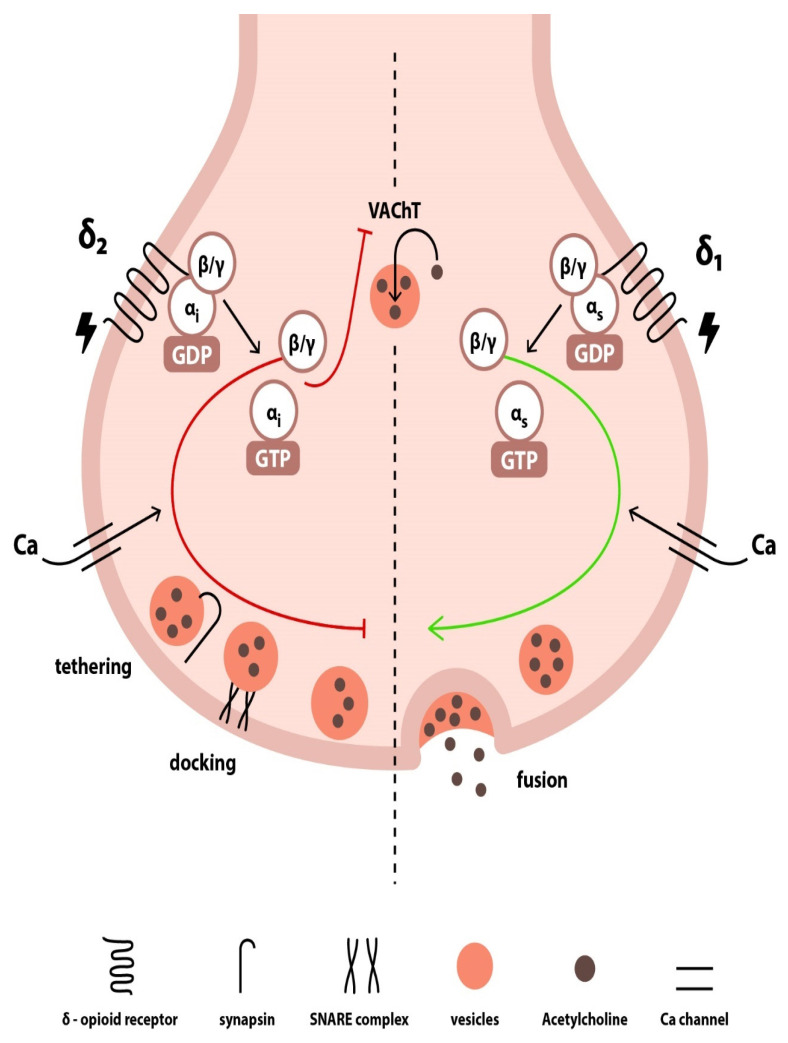
Modulation of presynaptic signaling in cholinergic nerve terminals by enkephalins. Enkephalins modulate presynaptic signaling in cholinergic nerve terminals innervating the sinoatrial node by acting on two functionally different subtypes of δ (delta)-opioid receptors (ORs), namely δ1-ORs and δ2-ORs. These two subtypes of δ-ORs are activated by enkephalins in a concentration-dependent manner, thereby resulting in bimodal effects on acetylcholine (Ach) release [27,29]. Low doses of enkephalins activate the δ1-OR, a stimulatory G protein-coupled receptor (Gs), which augments Ach release and induces vagotonic effects. On the contrary, higher levels of enkephalins activate the δ2-OR, an inhibitory G protein-coupled receptor (Gi), which inhibits Ach release and promotes vagolytic effects [27,28]. δ1-OR activation by enkephalins initially leads to the dissociation of the Gαs subunit from the Gβ/γ heterodimer. In turn, the dissociated Gαs subunit increases the activity of adenylyl cyclase and the production of cyclic adenosine monophosphate (cAMP), while the Gβ/γ subunit promotes calcium (Ca) entry into the nerve terminal (green arrow), thereby enhancing Ach release [38,39]. Concerning δ2-OR activation, the mechanism of action likely involves several pathways. Upon δ2-OR activation, the Gαi subunit reduces adenylyl cyclase activity, while the Gβ/γ subunit inhibits voltage-gated Ca channels (red arrow), through which Ca enters the nerve terminals to activate calcium sensor proteins and trigger the initiation of docking and fusion processes. The net result of decreased Ca influx is attenuated cholinergic transmission due to decreased Ach release in the synaptic cleft [28,31,32]. Moreover, the dissociated Gβ/γ subunit modulates the function of the soluble N-ethylmaleimide-sensitive factor attachment protein receptor (SNARE) complex, which is a protein apparatus embedded on both vesicular and plasma membranes that is actively involved in the docking and fusion of synaptic vesicles and subsequent neurotransmitter exocytosis [32]. The Gβ/γ subunit appears to interact with the SNARE complex mainly through competing with synaptotagmin (a Ca sensor protein that promotes the fusion of the vesicles with the plasma membrane) for binding sites on the SNARE complex, thus resulting in inhibition of Ach exocytosis [31,32,33]. In parallel, it is also possible that δ2-OR activation interferes with the function of synapsin, which has been implicated in the processes of tethering, docking, mobilization, and fusion of the synaptic vesicles. Under resting states, synaptic vesicles are reversibly tethered to the meshwork of the actin cytoskeleton by synapsin, which thereby acts by linking adjacent synaptic vesicles with each other and keeping them clustered and anchored in a distal reserve pool away from the plasma membrane. Apart from its tethering action, synapsin is also involved in the docking, post-docking, and fusion events. Upon phosphorylation of synapsin, synaptic vesicles are mobilized from the reserve pool, dissociate from the cytoskeleton and from each other, and move to the readily releasable pool, close to the synaptic cleft, where they are now free to fuse with the plasma membrane and release their content through exocytosis [34]. Accordingly, following δ2-OR activation, it could be postulated that the δ2-OR-coupled Gi protein inhibits synapsin phosphorylation and the downstream events, eventually resulting in reduced Ach release from the presynaptic cholinergic nerve terminal. Finally, δ2-OR signaling seems to modulate vesicular acetylcholine transporter (VAChT) turnover by inhibiting VAChT, which is a vesicular membrane-bound transporter that facilitates entry and storage of Ach into the synaptic vesicles. Due to VAchT inhibition, Ach cannot move into the synaptic vesicles and thus cannot be secreted in the synaptic cleft [29,35].

From a physiological perspective, several mechanisms that underlie OR-mediated neurotransmitter release in presynaptic nerve terminals have been implicated. Activation of the δ-ORs results in a conformational change of the receptor, which is propagated to a G stimulatory or inhibitory protein (depending on the nature of the δ-OR) and leads to the replacement of guanosine diphosphate (GDP) with guanosine triphosphate (GTP) on the Gα subunit. This latter phenomenon leads to the dissociation of the Gα subunit from the Gβ/γ heterodimer, both of which act thereafter on several downstream effector proteins and voltage-gated calcium (Ca) channels [30]. On this account, one main mechanism involves the inhibition of Ca channels by the dissociated Gβ/γ subunit of the δ-2 Gi protein-coupled OR, which leads to decreased intracellular Ca levels and subsequent suppression of neurotransmitter release since vesicle release is highly dependent on Ca levels. Contrariwise, dissociated Gβ/γ subunit of the δ-1 Gs protein-coupled OR stimulates Ca channel opening, thereby improving cholinergic neurotransmission. Another mechanism involves the direct binding of the OR-coupled Gβ/γ subunit to the soluble N-ethylmaleimide-sensitive factor attachment protein receptor (SNARE) complex of the vesicle fusion apparatus, which results in inhibition of vesicular membrane fusion. In this case, the Gβ/γ subunit interacts with synaptotagmin by means of competing for the same binding site on the SNARE protein complex, thereby impeding synaptotagmin from promoting vesicle fusion [31,32,33]. Moreover, Deo et al. [29] further reported that, in the canine sinoatrial node and atria, δ-ORs are colocalized with synapsin (a phosphoprotein that regulates acetylcholine release at the presynaptic level by modulating synaptic vesicle trafficking and availability for release), as well as with vesicular acetylcholine transporter (VAChT), which facilitates the entry of acetylcholine into the synaptic vesicles. The strong colocalization and the close association among δ-ORs, synapsin, and VAChT within the parasympathetic nerve terminals innervating the sinoatrial node and the atria further support the notion of presynaptic acetylcholine modulation by enkephalins in the cardiac tissue [29,34,35]. Interestingly enough, in contrast to postsynaptic signaling, which becomes saturated upon repetitive stimulation, presynaptic ORs resist desensitization even when repeatedly activated [36] via a mechanism called lateral receptor trafficking [31,37]. In this regard, lateral receptor trafficking in autonomic nerve terminals reflects an inherent ability of the enkephalinergic system to continuously modulate presynaptic neurotransmitter release. The presumed mechanisms underlying the modulation of presynaptic signaling by enkephalins in cholinergic nerve terminals are depicted in Figure 2.

### 3.3. Postsynaptic Effects

δ-ORs on myocardial sarcolemma can modulate the function of b (beta)-adrenergic receptors (ARs) located in the nearby vicinity through a process called cross-talk [40]. Enkephalins are co-released with catecholamines in the synaptic cleft during sympathetic stimulation, and it has been suggested that postsynaptic OR stimulation by enkephalins can blunt the responsiveness of adjacent b1-ARs to catecholamines, thereby attenuating AR-induced positive inotropic effects [40,41]. Both b1-ARs and ORs belong to the family of G protein-coupled receptors [30]. In the case of b1-ARs, the G protein is a stimulatory Gs protein, whereas ORs are usually coupled to an inhibitory Gi protein [40]. Upon b1-AR activation, a conformational change in the receptor leads to the exchange of GDP with GTP on the α subunit, which promotes the dissociation of the α subunit from the β/γ complex of the Gs protein [30,42]. This triggers a downstream signaling cascade, which involves the activation of adenylyl cyclase, the production of cAMP, and the activation of protein kinase A (PKA) [30]. In turn, PKA amplifies the Ca current through the LTCC channels on the T-tubules and augments the opening of the isoform 2 ryanodine receptors (RyR) with subsequent release of Ca from the sarcoplasmic reticulum (SR), thereby enhancing the excitation–contraction coupling and the effective initiation of cardiac contraction [43]. On the other hand, when enkephalin binds to the ORs, a Gi protein is activated, and the dissociated α subunit leads to the inhibition of adenylyl cyclase, which results in the decrease in the Ca transient, manifesting as negative inotropy [40].

As a matter of fact, ex vivo administration of Leu-enkephalin in isolated rat cardiomyocytes receiving norepinephrine depressed norepinephrine-driven contractility by interrupting the LTCC current and thus suppressing the rise in cytosolic Ca [41]. When the same authors compared the effects of δ-OR activation by Leu-enkephalin between norepinephrine-stimulated and non-stimulated rat hearts, they reported that Leu-enkephalin attenuated LV contractility and cAMP levels only in the group receiving adrenergic stimulation, whereas it had no effect on the basal contractility or cAMP levels of the control group. Therefore, the effects of enkephalins seem to be mediated by an antagonism between the OR and the b-AR signaling pathways [40]. Additionally, naloxone administration abolished the negative inotropic effects of Leu-enkephalin, as did the administration of a b2-agonist, which enhances contractility via a cAMP-independent signaling pathway [40]. These findings confirmed the existence of a cross-talk process between δ-OR and b1-AR (but not b2-AR) signaling, whereby b1-adrenergic effects are counteracted by δ-OR stimulation via a cAMP-dependent pathway. Interestingly, when an adenylyl cyclase activator was introduced to the cardiac tissue specimen to evoke an increase in LV systolic pressure, Leu-enkephalin successfully reversed the positive inotropic effect, but when second messengers downstream from the activation of adenylyl cyclase were introduced (such as the addition of a cAMP analog or the elevation of cytosolic Ca), Leu-enkephalin failed to suppress the positive inotropic action induced by the second messengers [41]. Hence, the inhibitory action of enkephalins appears to be restricted to the initial stages of the cascade, which involve the Gs phosphorylation and the activation of adenylyl cyclase, namely phenomena proximal to cAMP synthesis.

Other studies, however, have demonstrated the presence of additional OR signal transduction pathways, which operate independently of the b1-AR signaling pathway and seem to involve second messengers other than cAMP [44,45]. Ventura et al. reported that δ-OR and κ-OR stimulation exerts negative inotropic effects on rat ventricular myocytes by interfering with phosphatidylinositol turnover and leading to Ca depletion from the SR, as evidenced by an increase in inositol 1,4,5-trisphosphate (IP3) along with a decrease in the cytosolic Ca transient [44]. Since ORs are G protein-coupled receptors and IP3 is a well-known second messenger of the Gq protein signaling pathway, it appears that, upon OR activation by enkephalins, the dissociated Gaq subunit of the OR-coupled Gq protein activates phospholipase C, which in turn leads to the production of IP3 and diacylglycerol (DAG). IP3 opens specific Ca channels on the SR membrane by binding to its IP3 receptor, while DAG activates protein kinase C (PKC) [30]. Accordingly, the observed negative inotropic effects of Leu- and Met-enkephalin on the contractile function of rat cardiomyocytes seem to result from an increase in IP3 levels, which in turn leads to mobilization of SR Ca stores, progressive SR Ca depletion, and subsequent decreased cytosolic Ca transient [44].

On the other hand, in the study by Ela et al., selective stimulation of δ-OR resulted in the attenuation of the contractile force of isolated neonatal rat cardiomyocytes, which, however, was not related to alterations of cytosolic Ca transient, given that the amplitude of Ca influx and the concentration of cytosolic free Ca remained unchanged. Instead, it was reported that the negative inotropy was elicited by a reduction in the intracellular pH, which rendered the myosin filaments unresponsive to Ca [45]. Although the study used morphine, a non-selective OR agonist, the effect on pH alteration was abolished only when administering a δ-OR antagonist. In this case, the OR-mediated negative inotropy was shown to be effectuated by a pertussis toxin-sensitive Gi protein, while the signaling pathway seemed to involve the inhibition of PKC and of the sodium–hydrogen exchanger (Na^+^/H^+^ channel), eventually leading to the retention of H^+^ intracellularly [46].

**Figure 3:**
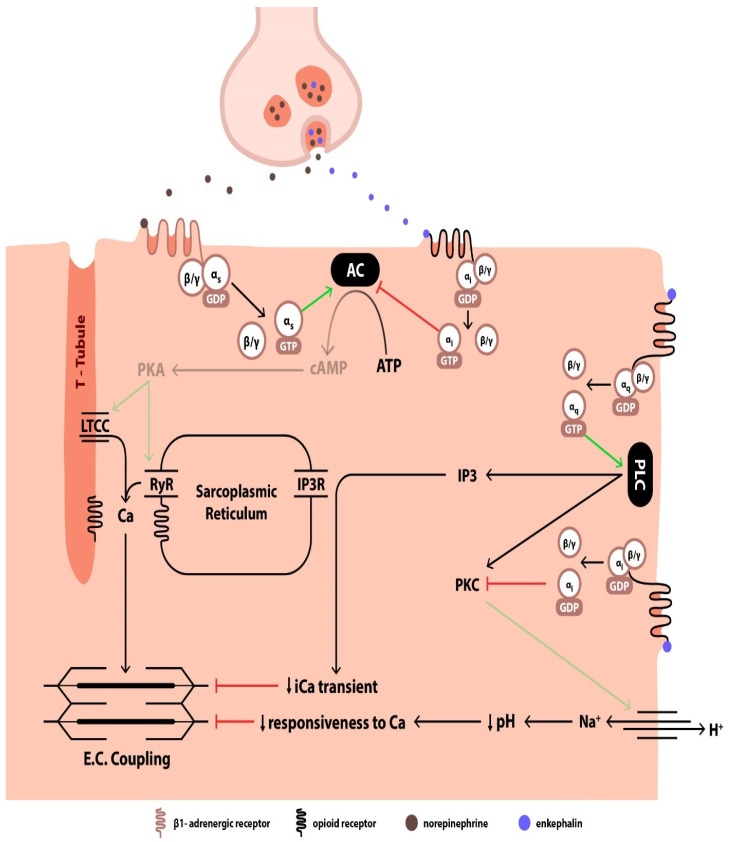
Modulation of opioid receptor-mediated postsynaptic signaling by enkephalins. On the postsynaptic membrane, the activation of the opioid receptor (OR) by enkephalins generates a sympatholytic effect via a cross-talk process between the OR and the beta 1 (b1)-adrenergic receptor (AR) [40]. Normally, upon norepinephrine binding to the b1-AR, a conformational change in the G protein-coupled receptor occurs, which leads to the dissociation of the Gαs subunit from the Gβ/γ subunit. This results in the sequential activation (green arrow) of adenylyl cyclase (AC) and protein kinase A (PKA), which in turn triggers the opening of both L-type calcium channels (LTCC) on the T-tubules and the ryanodine receptors (RyR) on the membrane of the sarcoplasmic reticulum (SR) [30,43]. Accordingly, both the amplification of calcium (Ca) influx through LTCC and the augmented release of Ca from the SR through RyR result in the increase in intracellular Ca levels and the subsequent stimulation of the excitation–contraction coupling [43]. During adrenergic stimulation, enkephalins are co-released with norepinephrine in the synaptic cleft and bind to the OR. Subsequent OR activation leads to the dissociation of the inhibitory Gαi subunit from the trimeric G protein. Thereafter, the Gαi subunit inhibits (red arrow) adenylyl cyclase and results in the reduction of all downstream messengers, thereby blunting the positive inotropic effects mediated by the b1-AR signaling [30,40,41]. It should additionally be noted that, apart from the postsynaptic membrane, ORs are also present in the T-tubule (in proximity with LTCC), as well as in the SR (colocalized with isoform 2 RyR) [5,6]. In parallel, OR activation appears to further inhibit excitation–contraction coupling via a non-b1-AR cross-talk manner, whereby enkephalins stimulate sarcolemmal δ-ORs of the Gq family [44]. In this case, dissociation of the Gaq subunit leads to the activation of phospholipase C (PLC) with subsequent synthesis of diacylglycerol (DAG) and inositol 1,4,5-trisphosphate (IP3). In turn, IP3 activates the IP3 receptor on the SR membrane and promotes Ca release from the SR [30,44]. Although initially, an increase in cytosolic Ca favors excitation–contraction coupling, it appears that enkephalins eventually deplete SR Ca stores and lead to a reduction in cytosolic Ca transient [44]. Additionally, DAG normally activates protein kinase C (PKC), which stimulates sodium–hydrogen (Na^+^/H^+^) antiporter to move Na^+^ inside the cell in exchange for H^+^. However, it seems that another alternative Gi OR-mediated pathway exists, which leads to the inhibition of PKC and the subsequent inhibition of Na^+^/H^+^ exchanger, thereby resulting in the reduction of intracellular pH and consequently in the decreased responsiveness of myosin filaments to Ca [45,46].

Figure 3 summarizes the postsynaptic signaling pathways that come into play following the activation of ORs by enkephalins.

## 4. Enkephalin-Mediated Regulation of Renal Function

Altered kidney function is another hallmark of HF, whereby continuous neurohormonal activation leads to inappropriate sodium and volume retention [1,47] and may be of significance when attempting to fully understand the enkephalinergic response to HF. Although the enkephalinergic modulation of renal function has not been studied in the context of HF, ORs are expressed in rodent [10] and human [16] renal tissue, and the association between OR agonism and diuretic effects has been well demonstrated. The diuretic effects involve neurally-mediated and neurally-independent pathways [48], which influence natriuresis and aquaresis, with the latter being more evident in human interventional studies [49,50,51].

In animal experiments, administration of selective OR agonists leads to modulation of urinary sodium excretion, which requires an intact renal innervation, while it also induces diuretic effects, which, however, remain unaffected after renal denervation and presumably involve suppression of the antidiuretic hormone (ADH) [48]. Regarding studies in human subjects, it has been demonstrated that stimulation of ORs leads to diuresis. Intravenous administration of FK 33–824, a Met-enkephalin analog, led to increased free water clearance in healthy subjects, accompanied by a simultaneous decrease in ADH levels. Thus, the diuretic effect was attributed to a central inhibition of ADH secretion and was abolished when an exogenous ADH analog was co-administered [49]. A similar study evaluated the urinary excretory effects of a different Met-enkephalin analog in relation to the subjects’ hydration status in order to explore the mechanistic nature of ADH inhibition. The administration of the Met-enkephalin analog inhibited the osmotically mediated ADH secretion after water deprivation challenge or hypertonic solution infusion. These effects were mediated presumably via δ-OR stimulation, and, interestingly enough, they were not reproduced in overhydrated subjects [50]. On the other hand, administration of selective κ-OR agonists increased urinary output and decreased urine osmolality without affecting ADH levels or urine electrolyte excretion [51], thereby suggesting a peripheral inhibition of ADH action.

## 5. Changes in the Enkephalin System in HF

Since the presence of ORs and proenkephalin is well documented in cardiac tissue, it is not surprising that HF induces changes in the enkephalin system, which in turn exerts several effects on the cardiovascular system. Indeed, in animal HF models, HF development has been associated with an upregulation in the relative abundance of ORs and enkephalins [5,6]. In experimental rat models of HF precipitated by overt hemodynamic load, δ-ORs [5] and κ-ORs [6], but not μ-ORs [17], increased after the development of HF. Moreover, higher expression of proenkephalin mRNA coupled with increased production of proenkephalin was reported in the left ventricle of rats with HF compared to controls [5].

In human subjects, Fontana et al. studied EOP plasma levels in 20 patients with acute heart failure (AHF), who were divided into two groups based on their clinical severity. Met-enkephalin was significantly increased in patients with AHF compared to healthy controls. Even more so, Met-enkephalin levels correlated positively with AHF severity. A positive correlation was also evident between Met-enkephalin and norepinephrine in patients with more severe forms of AHF, consistent with a higher degree of cross-talk between ORs and b1-ARs during sympathoadrenergic hyperactivity [52]. Of note, it was also reported that the enkephalinergic system affected atrial natriuretic peptide (ANP) levels in a variable manner. In patients with severe AHF, EOPs seemed to reduce ANP secretion in an effort to mitigate excessive noradrenergic stimulation. On the contrary, in patients with less severe AHF, characterized by moderate sympathetic activity, EOPs were found to exert a direct stimulatory effect on ANP secretion [52].

It could be argued that the enkephalinergic system seems to ‘adapt’ to HF by overexpressing ORs and by upregulating enkephalin peptides. The increase in the number of ORs on the sarcolemmal membrane, as well as the enhanced OR affinity, is considered to be a direct result of sympathetic overactivation, which is a dominant feature of HF. Yet, the same phenomena have also been observed in non-HF rats, which were subjected to pharmacological adrenergic agonism [53]. HF has been associated with an increase in δ-ORs and κ-ORs, both of which have been found to be distributed along the cytoplasmic membrane or be colocalized with voltage-gated LTCC and RyR, suggesting a direct effect of EOPs on Ca handling [5,6]. The adaptive mechanisms of the enkephalinergic system in HF, wherein ORs and enkephalins are upregulated, seem to come into contradiction with the catecholaminergic pathophysiology of HF, where ARs are downregulated and become desensitized to the overt adrenergic stimulation [54].

Finally, it is important to note that OR activation has been shown to influence the arrhythmogenic risk in diseases that contribute to the development of HF, such as myocardial infarction. Studies have extensively investigated OR activation in the context of ischemic reperfusion injury, with findings suggesting that agonism of both κ-ORs and δ-ORs exert cardioprotective action by increasing the threshold for arrhythmia development [55,56]. As a matter of fact, enkephalins appear to attenuate epinephrine-induced arrhythmias via δ1-ORs [57]. The underlying OR-mediated antiarrhythmic mechanisms are similar to the postsynaptic mechanisms previously described for the EOP actions on excitation–contraction coupling. In particular, they seem to involve either a c-AMP-dependent pathway arising from the cross-talk between Gi protein-coupled OR and b1-AR [58] or a PKC-related phosphatidylinositol pathway [55,59]. Regarding the former signaling pathway, Xu et al. studied the modulating effects of U50,488H (a selective κ-OR agonist) on the arrhythmogenic action of norepinephrine and confirmed that U50,488H mitigated the arrhythmogenic effects of norepinephrine by inhibiting the norepinephrine-potentiated cAMP synthesis and the resultant intracellular Ca oscillations responsible for the arrhythmias. On the other hand, U50,488H failed to demonstrate any protective effects against arrhythmias in the absence of adrenergic stimulation with norepinephrine [58]. On this account, the b-blocking activity induced by OR stimulation appears to mimic the actions of class II antiarrhythmic drugs. With regard to the phosphatidylinositol signaling pathway, κ-OR agonism activates PKC and potentiates mitochondrial ATP-sensitive potassium channels, which play a key role in electrical stability by protecting the cardiomyocyte from the development of energy deficiency during ischemia and by preventing Ca overload [55,59,60]. However, alternative OR-independent pathways have also been described. At higher doses, κ-OR agonists have been found to exert direct actions on cardiac ion channels without interacting with myocardial ORs. These direct electrophysiological actions may include PR interval prolongation and QRS widening, namely changes indicative of sodium (Na) channel blockade, which resemble the actions of class I antiarrhythmic drugs. Additionally, κ-OR agonists may increase the refractory period and cause QT prolongation by exerting direct blocking activity on potassium (K) channels, thereby mimicking the action of class III antiarrhythmic drugs [55,56,61].

## 6. Modulation of the Endogenous Opioid System in HF

In order to draw conclusions about the EOP actions, several investigators have studied the effects of modulation of the EOP system by administering OR agonists or inhibitors. In a hamster model of hypertrophic cardiomyopathy and HF, ex vivo perfused working hearts were infused with selective μ-OR, δ-OR, or κ-OR agonists. In the failing hearts, δ-OR and κ-OR agonism led to significant depression of cardiac contractility in a dose-dependent manner, whereas administration of a selective μ-OR agonist failed to induce any negative inotropy and lusitropy when given at similar doses. The cardiac depressant effects of δ-OR and κ-OR agonists were found to be elicited via a Gi-mediated inhibition of cAMP and a decrease in Ca transient [62].

Liang et al. studied the in vivo hemodynamic effects of nalmefene, a non-selective OR antagonist, in an experimental canine HF model. Nalmefene improved cardiac output and LV performance in dogs with HF, in contrast to sham-operated dogs, in which hemodynamics remained unaffected by the drug. Interestingly, pre-treatment with b-blockers abolished the beneficial effects of OR antagonism in dogs with HF [63], suggesting that the positive inotropic effects of OR antagonism were mediated through b-AR signaling. This in vivo demonstration of b-AR-mediated opioid antagonism further corroborates the concept of existing interactive cross-talk between OR and b1-AR signaling pathways. Likewise, intravenous administration of naloxone (a non-selective OR antagonist) improved cardiac inotropy and systemic blood pressure in dogs with both left- and right-sided HF [64,65]. To distinguish which specific OR subtype mediates the salutary effects exerted by OR antagonists on hemodynamics and cardiac contractility, Imai et al. investigated the cardiovascular effects of selective μ-OR or δ-OR antagonism in dogs with right HF and elevated EOPs. They found that only δ-OR, and not μ-OR, inhibition mimicked naloxone effects by producing beneficial hemodynamic responses and concluded that cardiovascular regulation by opioids in HF is mediated via δ-ORs [65].

In humans with HF and elevated EOPs, few studies have assessed the hemodynamic effects of OR antagonism. In patients with AHF, intravenous administration of naloxone increased blood pressure and heart rate without affecting central venous pressure, while it also led to a hormonal upregulation of norepinephrine and ANP. These changes were evident only in the more severe HF group, whereas, in patients with less severe HF, naloxone actually decreased ANP levels while it had no effect on norepinephrine levels and hemodynamic parameters. This suggests that naloxone effects depend on the intensity of the sympathetic activity. As discussed in the previous section, in the most severe cases of AHF, characterized by overt sympathetic stimulation, EOPs exert inhibitory effects on norepinephrine and ANP release, which are abolished by naloxone administration, thereby resulting in norepinephrine and ANP hypersecretion. Such an effect is not observed in the less severe AHF cases characterized by moderate sympathoadrenergic activity [52]. Moreover, a small-scale study evaluated the acute hemodynamic effects of naloxone in 8 patients suffering from stable chronic HF, with a mean LV ejection fraction (LVEF) of 19%, and New York Heart Association (NYHA) class III-IV. The patients received incremental doses of naloxone, and several hemodynamic parameters were recorded through right heart catheterization at baseline, immediately before each increase in naloxone dose, and after the end of the infusion. Hemodynamic parameters, such as right atrial pressure, pulmonary capillary wedge pressure, right and left ventricular stroke work index, pulmonary and systemic vascular resistance, and cardiac output were among the measured hemodynamic variables; yet, none showed any significant changes after naloxone administration compared to baseline values [66]. Likewise, in another small subgroup of patients from the same study with NYHA class II-III HF, naloxone failed to increase exercise capacity or alter any parameters of cardiovascular and respiratory performance during the cardiopulmonary exercise test [66].

## 7. PENK119–159 in HF

The intricate role of enkephalins in patients with HF remains elusive. Since Fontana et al. demonstrated a direct relationship between EOPs and neurohormonal modulation in patients with severe AHF [52], little progress has been made regarding their role in the setting of HF. Nonetheless, recent observational studies on human cohorts suggest a possible interplay between the endogenous opioid and cardiovascular system since there is emerging evidence about existing correlations of PENK_119–159_ levels with HF severity, mortality, and cardiorenal dysfunction. While, in healthy subjects, the mean (SD) concentration of PENK_119–159_ has been reported to be 46.34 (14.60) pmol/L [4], in a recent systematic review of HF studies, the cut-off level above which PENK_119–159_ values in patients with HF were considered to be high varied among the studies, ranging from 67 to 83 pmol/L [2].

In 2014, Ng et al. conducted the first study on the prognostic role of PENK_119–159_ in 1141 patients with acute myocardial infarction. In this observational single-center study, PENK_119–159_ levels upon admission correlated significantly with age, clinical signs (heart rate and diastolic blood pressure), renal impairment [as reflected by estimated glomerular filtration rate (eGFR)], and markers of cardiac dysfunction, namely LVEF and N-terminal pro-brain natriuretic peptide (NT-proBNP) levels. Higher baseline PENK_119–159_ levels were independently associated with an increased risk for major adverse cardiac events (MACE) at two years, defined as a composite of death, recurrence of myocardial infarction, and HF hospitalization. Sequential measurements of PENK_119–159_ during the following days of admission did not offer any statistically significant correlation with risk for MACE. Interestingly, patients were divided into four groups based on PENK_119–159_ values (<39.9, 40.0–55.5, 55.6–83.2, and >83.3 pmol/L) and it was found that patients at the fourth quartile (PENK_119–159_ > 83.3 pmol/L) experienced more MACE. Additionally, measurement of PENK_119–159_ offered added predictive value to the established Global Registry of Acute Coronary Events (GRACE) risk score for the prediction of death and/or recurrent myocardial infarction at 6 months. Moreover, PENK_119–159_ levels below 48.3 pmol/L were defined as a cut-off value for the identification of low-risk patients, whereas values over 91 pmol/L were indicative of high-risk patients [67].

Following this initial observation, further research focused on the predictive role of PENK_119–159_ in the context of HF. Eventually, there have been several reports about an existing association between PENK_119–159_ levels and parameters of cardiac dysfunction in patients with chronic or acute HF. In two studies that enrolled patients with chronic HF, PENK_119–159_ levels were significantly correlated with the severity of HF, lower LVEF [68], higher NYHA class, and higher NT-proBNP levels [69]. Similarly, in patients with AHF, it was found that PENK_119–159_ was significantly associated with brain natriuretic peptide (BNP)/NT-proBNP levels [69,70,71,72], NYHA class [72] and echocardiographic variables of diastolic dysfunction (E/e’) [71].

Furthermore, a relationship between PENK_119–159_ and kidney function was reported in both acute [69,70,71,72,73,74] and chronic [68,69] HF, in view of the fact that PENK_119–159_ was significantly associated with various markers of renal dysfunction, such as elevated serum creatinine, urea, albuminuria, and low eGFR. Since PENK_119–159_ is a small peptide undergoing free glomerular filtration, these findings could mirror the presence of defective glomerular filtration, which leads to the accumulation of PENK_119–159_ in the plasma of patients with HF. In this regard, PENK_119–159_ could serve as an early and reliable indicator of renal dysfunction. In addition, the association of PENK_119–159_ levels with markers of tubular damage has been investigated, yet with conflicting results. In the study by Matsue et al., no significant correlation was found between PENK_119–159_ levels and urinary tubular markers, such as Kidney Injury Molecule 1 (KIM-1), N-acetyl-β-D-glucosaminidase (NAG) and Neutrophil Gelatinase-Associated Lipocalin (NGAL) [69]. Contrariwise, in the study by Emmens et al., plasma NGAL turned out to be one of the strongest predictors of PENK_119–159_ levels, while urinary KIM-1 also showed significant correlation with elevated PENK_119–159_ values [75]. Moreover, in patients with AHF complicated with acute kidney injury, levels of serum NGAL were found to be elevated in accordance with PENK_119–159_ levels [73,76]. A possible explanation of the observed discrepancy between PENK_119–159_ and NGAL could be that HF populations were different among studies. Indeed, the study by Matsue et al. included patients with chronic HF, whereas the other studies that reported a correlation between PENK_119–159_ and NGAL involved patients with AHF. It could be assumed that the observed association of PENK_119–159_ and NGAL in the acute setting may be attributed to the fact that patients with acute decompensated HF run a greater risk of developing more severe cardiorenal syndrome with subsequent acute kidney injury and overt tubular damage, due to exaggerated renal hypoxia resulting from a higher degree of congestion.

In an attempt to further elucidate the involvement of PENK_119–159_ in the development of cardiorenal derangement, several studies have focused on the role of PENK_119–159_ as a predictor of worsening renal function (WRF) in patients with HF. Although PENK_119–159_ levels exhibited limited prognostic utility for WRF in a study including a cohort of patients with AHF [69], other studies have reported a positive correlation between PENK_119–159_ levels and WRF. Higher levels of PENK_119–159_ on admission were correlated with deteriorating renal function [70,76] and proved to be a significant independent predictor of WRF in the short term, with areas under the curve (AUCs) ranging from 0.652 to 0.899 [69,70,76]. In the study by Emmens et al., elevated PENK_119–159_ levels were also associated with WRF, even after adjusting for other markers of kidney injury, such as plasma and urinary NGAL, urinary KIM-1, and urine albumin-to-creatinine ratio. It should be noted that this result should be viewed as a long-term outcome, considering that renal dysfunction was examined after a 9-month period [75]. Moreover, in patients with acute decompensated HF, high levels of PENK_119–159_ were independent predictors of the development of cardiorenal syndrome type 1 [76]. Likewise, in a multicenter cohort study of patients with cardiogenic shock, elevated levels of PENK_119–159_ on admission were independently associated with the occurrence of acute kidney injury within 48 h. In patients with end-stage renal disease undergoing hemodialysis or peritoneal dialysis, however, the association between PENK_119–159_ levels and HF, LVEF, and natriuretic peptides seems to weaken, and therefore, PENK_119–159_ measurement may be of limited significance in this specific population [77].

The principal aim of human observational studies was to explore the prognostic impact of PENK_119–159_ in terms of morbidity and mortality [70,71,73,74,76]. In the study by Matsue et al., although PENK_119–159_ was initially associated with clinical outcomes (all-cause mortality at 6 months, HF rehospitalization within 60 days, death or rehospitalization due to cardiovascular or renal causes at 60 days) in the univariable analysis, its prognostic ability was subsequently lost for all outcomes when other established prognostic markers were introduced in the multivariable model [69]. Nevertheless, multiple other studies have underscored the valuable role of PENK_119–159_ in predicting short-term [70,76] as well as long-term outcomes [70,71,74]. As a matter of fact, in an observational multicenter cohort study of 1908 patients with AHF, it was shown that PENK_119–159_ levels could yield prognostic information on cardiovascular morbidity and all-cause mortality, both in the short term (3 and 6 months) and in the long term (1-year). Of note, PENK_119–159_ levels could also aid in the risk stratification of patients with AHF since levels below 133.3 pmol/L were associated with a low in-hospital mortality rate (2.1%), whereas levels above 211.3 pmol/L signified increased mortality (13.1%) [70]. Similar results were reported in another multicenter study of patients with acute decompensated HF and preserved LVEF. In this study, PENK_119–159_ could independently predict all-cause mortality or rehospitalization for HF at 2 years, while higher values were associated with worse outcomes and poorer prognosis [71]. Moreover, in another observational cohort study including 530 patients with AHF from Sweden and Italy, PENK_119–159_ levels were associated with in-hospital and 1-year all-cause mortality, with increasing values conferring a higher mortality risk; yet, PENK_119–159_ was not found to be associated with the length of hospital stay or the risk for HF rehospitalization [74]. In line with the above, a prospective observational study by Zhao et al. outlined the importance of PENK_119–159_ as an independent predictor of adverse outcomes, including HF readmission or all-cause death at 90 days [76], while, in another study including two large cohorts of patients with HF, elevated PENK_119–159_ levels were found to be significantly associated with higher all-cause mortality, but not with an increased risk of HF readmission [72]. Interestingly, in patients with cardiogenic shock, PENK_119–159_ levels were independently and strongly predictive of 90-day mortality only when measured at 24 h after admission [73], in contrast to previous studies [70,71,72,74,76], in which the baseline levels of PENK_119–159_ upon admission were found to carry prognostic value in terms of predicting the risk of adverse events.

## 8. Discussion

In patients with HF, the levels of both active enkephalins and their surrogate PENK_119–159_ are elevated and are associated with a dismal prognosis [2,52]. Notwithstanding the intriguing findings, there is still a gap of knowledge between current data on observational human studies and prior experimental evidence regarding the role of endogenous opioids in cardiovascular function during physiological and HF states that needs further elucidation.

Sympathetic overstimulation is a hallmark of HF decompensation and is more evident in patients with reduced LVEF. Initially, adrenergic overdrive acts as a compensatory mechanism that aims at counteracting the diminished cardiac output. The activation of the sympathetic limb of the ANS mounts a direct cardiostimulatory response, promotes catecholamine release from the adrenal medulla, and increases peripheral vascular resistance, leading to a reduction in renal perfusion. As a result, renin is secreted by the kidney and activates the renin–angiotensin–aldosterone system (RAAS), leading to aldosterone secretion by the adrenal medulla. Collectively, these actions are initially intended to restore cardiac output by producing favorable hemodynamic effects and by promoting sodium and water retention in the renal system, but they eventually become maladaptive and exert deleterious effects on cardiovascular and renal function [47,78]. The underlying pathophysiological mechanism that links sympathetic hyperactivity and impaired cardiac contractility probably lies in the dysfunction of RyR2 caused by excessive adrenergic signaling. In turn, RyR2 dysfunction results in a leaky SR, characterized by both sustained release of Ca and inappropriate Ca reuptake from the SR. The net result of the SR dysfunction is the depletion of intracellular Ca stores on the one hand and persistently high cytosolic Ca concentrations during both phases of the cardiac cycle on the other hand [79]. These perturbations manifest as derangements in the excitation–contraction coupling, defective diastolic relaxation, and systolic and diastolic ventricular dysfunction [79], eventually leading to desensitization of ARs [78]. In parallel with sympathetic hyperactivity, vagal downregulation is another prominent feature in patients with HF, which further exacerbates the imbalance between the sympathetic and parasympathetic CNS arm, ultimately leaving the sympathetic overdrive unopposed [47,80].

**Figure 4:**
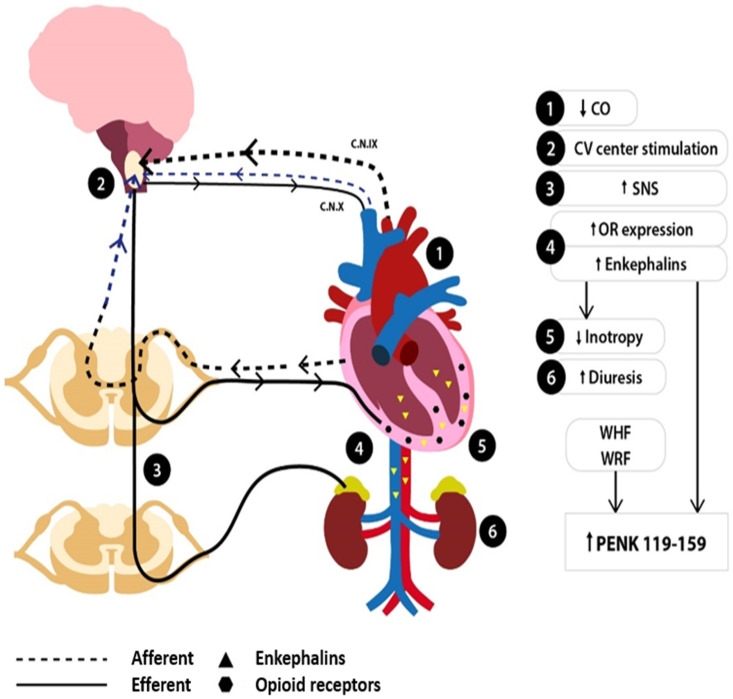
The interplay between neurohormonal and enkephalinergic overactivity in heart failure. Signals relating to the decreased cardiac output (CO) and the subsequent cardiac volume overload are correspondingly sensed by the aortic and carotid bodies on the one hand and by the cardiac sensory nerves on the other and are then relayed via the IX and X cranial nerves (C.N.IX and X), as well as via the thoracic spinal sensory tracts, respectively, to the cardiovascular (CV) center in the medulla oblongata. After all inputs are integrated and processed in the brainstem, the appropriate response arises, which is then conveyed through the efferent pathways to a variety of organs in order to maintain a certain degree of homeostasis [23]. In the setting of heart failure (HF), the net result is the adrenergic stimulation of the pre- and post-ganglionic sympathetic fibers, which innervate the cardiac, vascular, renal, and adrenal medullary tissues [47]. Simultaneously, an enkephalinergic response is mounted, which is characterized by an overproduction of enkephalins, coupled with overexpression of opioid receptors (OR), in an attempt to combat the underlying sympathetic hyperactivity. The upregulation of the enkephalinergic system blunts the b-adrenergic-mediated increase in cardiac contractility [40,41] in an effort to decrease cardiac workload and counteract the effects of increased sympathetic tone while it also stimulates renal diuresis [49] in order to achieve volume offloading. As HF progresses and renal deterioration ensues, plasma levels of PENK_119–159_ increase as a result of both enkephalin overproduction and decreased glomerular filtration. Although the counterregulatory mechanisms of the activated enkephalinergic system are initially intended to protect the cardiovascular system from the HF-driven sympathetic surge, they eventually become maladaptive, leading to further decline in cardiac function and renal deterioration. Abbreviations: C.N. = cranial nerve; CO = cardiac output; CV = cardiovascular; HF = heart failure; OR = opioid receptors; PENK = proenkephalin; SNS = sympathetic nervous system; WHF = worsening heart failure; WRF = worsening renal function.

On the other hand, the enkephalinergic system is recruited to mitigate overt neurohormonal activation [52]. Enkephalin levels increase within the myocardial tissue and the cholinergic and adrenergic ANS cardiac fibers [10,19,21], as well as in the periphery [52], where they are co-released with catecholamines from the adrenal medulla in the circulation [15,22]. Concomitantly, an upregulation of OR myocardial expression occurs [5,6]. The enkephalinergic response exerts cardiodepressive effects on the myocardium to attenuate the adrenergic-mediated positive inotropic effects [40,41] and promotes diuresis to achieve volume offloading [49]. The levels of the inactive peptide PENK_119–159_ may increase due to the continuous enkephalinergic activation and the reduced glomerular clearance once renal deterioration occurs. A simplified illustration of the events following HF development, along with the interplay between neurohormonal and enkephalinergic overactivity in HF, is depicted in Figure 4.

From a pathophysiological standpoint at the molecular level, enkephalins regulate cardiovascular function through three principal mechanisms. The first one involves the CNS [8], while the remaining two mechanisms antagonize sympathetic triggering either at the level of the nerve terminal (presynaptic level) or at the postsynaptic membrane. At the presynaptic level, a multimodal modulation of neurotransmitter release takes place. Indeed, enkephalins inhibit norepinephrine release from adrenergic nerve terminals [25], whereas in cholinergic nerve terminals, enkephalins may lead to either inhibition or stimulation of acetylcholine release [27]. The final effect on the cholinergic nerve terminals depends on the OR subtype involved, which in turn is contingent on the enkephalin levels, with low enkephalin levels exerting vagotonic effects and high levels acting vagolytically. Such a finding could suggest an initial vagotonic enkephalinergic response, which progresses to a vagolytic one once elevated enkephalin states are established, as is the case in severe HF or cardiogenic shock. At the postsynaptic level, enkephalins hinder b1-AR signaling by inhibiting adenylyl cyclase activation, thus blunting adrenergic effects [40,41]. Overall, enkephalin upregulation in HF facilitates a compensatory response to attenuate the underlying b-adrenergic overstimulation, essentially mimicking the effects of the protective compensatory downregulation and desensitization of b-ARs observed in patients with HF [47,78]. Furthermore, additional OR signaling pathways, leading either to the depletion of SR Ca stores or to cytosolic acidosis, participate in the cardiodepressive effects [44,45]. Moreover, enkephalins seem to possess autocrine and paracrine properties, as cardiomyocytes may also synthesize enkephalins [10,21], which are presumably capable of acting directly on the myocardium and the adjacent autonomic nerve terminals.

In the clinical setting, the enkephalinergic cardiodepressive effects highlighted in the experimental studies are corroborated by a constant clinical finding among most human studies, namely the association of enkephalins (or their surrogate PENK_119–159_) with subjective (NYHA class), clinical (lower blood pressure), and biochemical (higher natriuretic peptides, higher norepinephrine levels) parameters of HF severity [52,70]. Furthermore, the use of opioid antagonists has been shown to reverse the enkephalin-induced cardiodepressive effects and to improve the hemodynamic status of AHF patients [52]. Until, however, further research is undertaken in this direction, the primary clinical utility of PENK_119–159_ lies in its prognostic significance in terms of predicting adverse outcomes such as WRF, mortality, and HF readmission.

PENK_119–159_ has been identified as an independent predictive factor for the development of short- (within 5 days) [70,73,74] or long-term (at 9 months) [75] renal deterioration, even after extensive multivariate adjustment and despite heterogeneities in WRF definition across studies. Notably, PENK_119–159_ levels inversely correlate with glomerular filtration rate in patients with both acute and chronic HF [69,70,74,75,76]. Compared to serum creatinine’s inherent delay to increase, PENK_119–159_ exhibits different kinetics and earlier elevations in the setting of acute kidney injury, thus rendering this biomarker ideal for identifying real-time renal injury. Indeed, serial assessments of PENK_119–159_ levels in AHF patients, combined with the evaluation of PENK_119–159_-to-creatinine (P/C) ratio on admission and post-treatment, reveal that there is a substantial difference in the kinetics of PENK_119–159_ and creatinine in the setting of WRF. Compared to patients who had not developed WRF, those with WRF had higher baseline levels of PENK_119–159_ on admission, which further increased post-treatment. More importantly, in patients with WRF, their P/C ratio was initially elevated and decreased significantly with time, whereas in patients without WRF, their P/C ratio remained unaltered. The temporal pattern of change in the P/C ratio, which was observed only in patients with WRF, is compatible with a delayed, disproportional PENK_119–159_ rise in creatinine levels [70]. Moreover, baseline PENK_119–159_ levels are strongly correlated with both markers of glomerular dysfunction (creatinine, albuminuria) and tubular injury (KIM-1 and NGAL). Even more so, PENK_119–159_ has been shown to outperform them in predicting WRF by exhibiting superior diagnostic accuracy [75]. Zhao et al. further confirmed the role of PENK_119–159_ as a robust predictor of cardiorenal syndrome type 1, with an AUC of 0.9, while the reference biomarker NGAL failed to reach statistical significance [76]. The association with tubular injury markers was evident exclusively in studies comprising AHF patients with either de novo or worsening HF [75,76], whereas in the study by Matsue et al., no such association was observed [69]. Although it is difficult to draw firm conclusions that would explain this discrepancy, it could be argued that the study by Matsue et al. was retrospective in nature and consisted of two arms (patients with chronic and acute HF), with one arm being limited to a small sample of patients with stable chronic HF, presenting with reduced LVEF and mild renal impairment [69]. Although PENK_119–159_ is an independent factor for the development of WRF in different populations of patients with HF [70,73,74,75,76], it is not yet clear whether its implementation into WRF models containing clinical and laboratory variables improves their predictive value or not [70]. A recent consensus statement supports the use of PENK_119–159_ as a biomarker in acute kidney injury [81], but its role, specifically in HF-induced WRF (cardiorenal syndrome type 1 and 2), still remains elusive.

Additionally, PENK_119–159_ may also offer prognostic value as an independent predictor of both in-hospital [70,74] and long-term mortality [70,73,74,75], as well as HF readmission [76]. Net reclassification improvement analysis demonstrated that the addition of PENK_119–159_ to either a variety of clinical risk scores [70] or clinical models incorporating natriuretic peptides [71] enhanced risk stratification of AHF patients with regard to in-hospital mortality [70] and the composite endpoint of death/HF rehospitalization [71]. Interestingly, PENK_119–159_ retains its prognostic performance in HF patients, regardless of their EF [71] or stage of renal disease, with the sole limitation being end-stage renal disease [77].

Although studies confirm the prognostic clinical utility of PENK_119–159_, this biomarker has not shown promising results in predicting the risk of new-onset HF [72], nor has it been studied as a therapeutic target or as a tool for guiding treatment. Furthermore, heterogeneities in study design may be partially responsible for some contradictory results, given that variations in enrollment settings, patient characteristics, and timing of PENK_119–159_ measurement may affect PENK_119–159_ levels and their association with outcomes. Moreover, discordance has been noted among trials with respect to the reported cut-off values of PENK_119–159_. The different cut-offs may reflect differences in the clinical phenotypes of patients with HF. Indeed, the Cardshock study, which focused only on patients presenting with cardiogenic shock, reported that the optimal PENK_119–159_ cut-off at baseline was >84.8 pmol/L for predicting acute kidney injury, whereas the cut-off level of PENK_119–159_ at 24 h for predicting 90-day mortality was much higher (>105.7 pmol/L) [73]. Ng et al. reported that a cut-off <133.3 pmol/L was associated with low in-hospital mortality in patients with AHF, whereas a cut-off >211.3 was predictive of a high mortality rate [70]. These reported cut-offs are much higher than the 67–83 pmol/L range, which has been recently proposed as the definition of high plasma PENK_119–159_ levels in HF patients, irrespective of their clinical phenotype, according to a systematic review by Siranart et al. [2].

Since the publication of PENK_119–159_ studies, novel therapies have emerged for patients with HF and reduced LVEF, including sacubitril–valsartan, which is a regimen containing a neprilysin inhibitor [82]. Given that enkephalins are cleaved by several endopeptidases, including neprilysin, it is plausible that the inhibition of neprilysin may augment the enkephalinergic response by allowing the biologically active EOPs not only to achieve higher concentrations but also to circulate in the plasma for longer periods of time. In light of this, a study was performed in patients with HF and reduced LVEF who were receiving sacubitril–valsartan in order to investigate whether the initiation of a neprilysin inhibitor affects the dynamics of enkephalins, which are known substrates of neprilysin. To this end, plasma levels of PENK_119–159_ were measured at 0, 1, and 2 years, and overall analysis showed that PENK_119–159_ levels demonstrated a slight upward increase at 1 and 2 years of follow-up compared to baseline values. However, in paired sub-analysis, this elevation did not remain statistically significant at 2 years [83]. Notwithstanding, it needs to be emphasized that these findings for PENK_119–159_ cannot be extrapolated to the biologically active enkephalins, considering the fact that PENK_119–159_ actually serves as a surrogate marker of enkephalin synthesis, whereas the neprilysin inhibitor seems to influence the kinetics of enkephalins and not their production [83].

Based on the enkephalinergic hemodynamic effects, it would be intriguing to consider the endogenous opioid system as a promising target for future therapy. OR antagonists have demonstrated favorable hemodynamic effects in patients with AHF, but their safety and association with mortality have not been adequately studied. One should bear in mind that even long-standing therapeutic approaches, which prevailed in the past, have been debated in recent years, such as morphine administration in patients with AHF, given that contemporary studies indicate that morphine increases the risk of long-term mortality and invasive ventilation [84]. Hence, caution is required when investigating potential future therapeutic schemes based on OR antagonism since the benefits of administering OR-modulating agents are still indeterminate.

## 9. Conclusions

In the setting of HF, the synthesis of enkephalin peptides and opioid receptors is upregulated as a means to counterbalance the underlying sympathetic overdrive. Notably, biologically active enkephalins exert their effects mainly upon adrenergic overstimulation, considering that exogenous enkephalin administration fails to diminish contractility in the absence of a hyperadrenergic state. This may be explained by the principal action of enkephalins, which fundamentally function by impeding b1-AR hyperactivity [40,41]. In addition, the protective role of enkephalinergic upregulation in HF is further supported by the offloading effects of enkephalins through diuresis [49]. Accordingly, it could be speculated that the modulation of the enkephalinergic system in HF follows a similar pattern to the HF-induced activation of the adrenergic system, which initially acts as a protective compensatory mechanism but becomes maladaptive at later stages, resulting in deleterious effects as HF progresses. Indeed, it seems that the compensatory actions of enkephalins resemble the effects of the therapeutic manipulations targeting at b-blockade [23]. The parallel elevations of norepinephrine, mature enkephalins, and inactive enkephalin peptide PENK_119–159_ in HF, coupled with their positive correlations with increased mortality, point toward an interaction between the adrenergic and the enkephalinergic signaling pathway; yet, further research is warranted in order to unfold any causal relationship between these two distinct pathways that come into play in patients with HF.

For the time being, the potential clinical utility of PENK_119–159_ lies in its prognostic role. Compared to conventional biomarkers, PENK_119–159_ seems to confer an added value in predicting WRF, mortality, and HF readmissions, which renders it both an early detection and a risk-stratification biomarker. However, in order to establish clear cut-offs for the clinical endpoints, further research is required in patients with both acute and chronic HF, as well as in subjects presenting with different clinical phenotypes of AHF. Meanwhile, the introduction of point-of-care assays will allow the bedside evaluation of PENK_119–159_ [85] and will most likely facilitate the conduction of well-designed interventional studies since monitoring of real-time PENK_119–159_ changes will become feasible. The strong association of PENK_119–159_ with both WRF and mortality set the grounds for further research regarding its clinical utility in monitoring treatment, which remains a clinical field where other biomarkers have failed to show any clinical benefit. Undoubtedly, deciphering the exact pathophysiological mechanisms that lead to the upregulation of PENK_119–159_ in HF is crucial in order to develop a more consistent patient-centered approach and effectively guide therapeutic strategies in patients with HF toward achieving better survival.

## Data Availability

No new data were created or analyzed in this study.
